# Supplementary material for: Genetic Spectrum of Syndromic and Non-Syndromic Hearing Loss in Pakistani Families
Source: Genes (Basel). 2020 Nov 11;11(11):1329. doi: 10.3390/genes11111329 (PMC7709052; doi:10.3390/genes11111329)
Supplement: Supplementary file 1 [file genes-11-01329-s001.zip › Supplementary Files/Figure S1_Figure S2.pptx]

## Slide 1
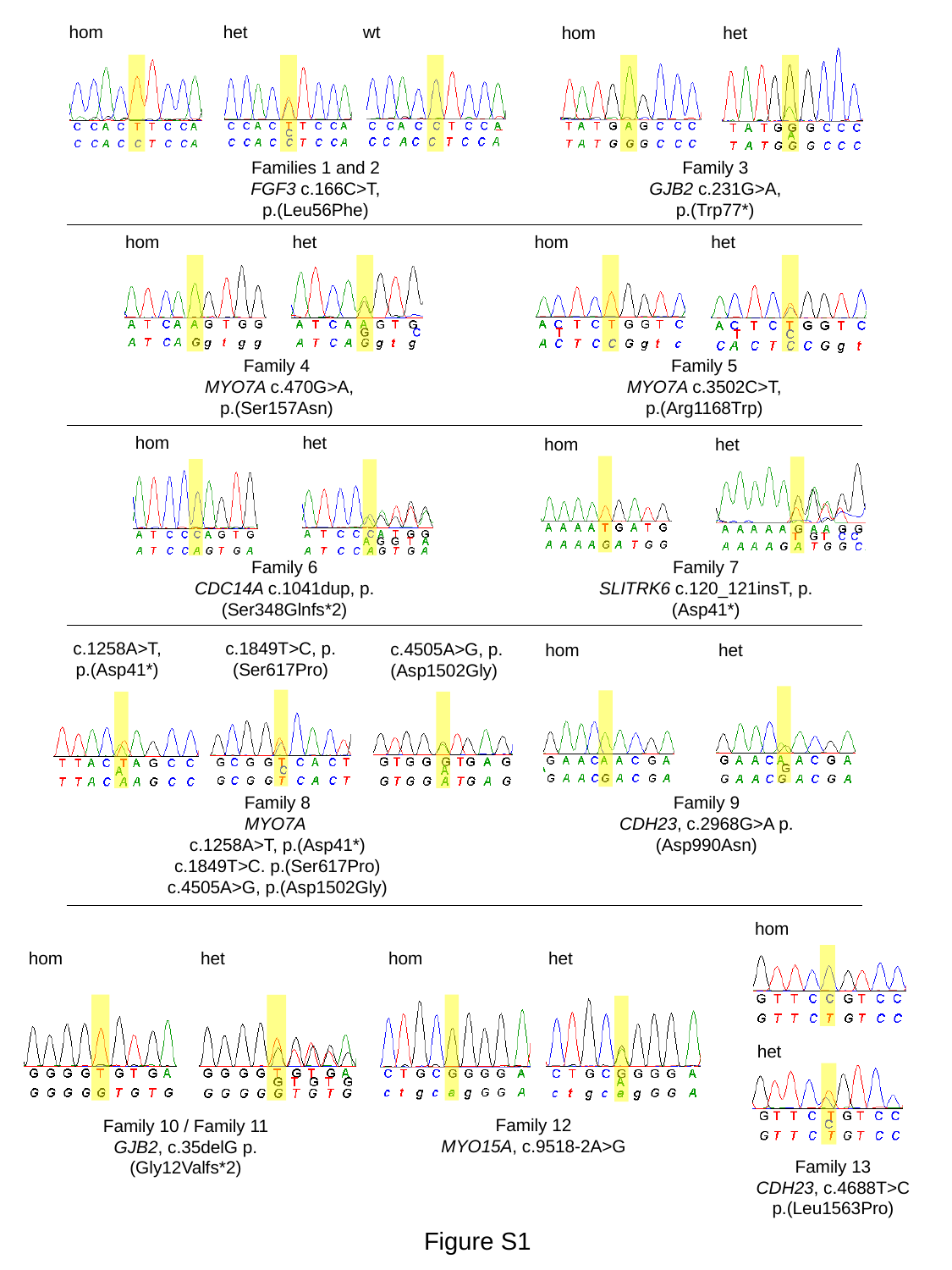

hom
het
Families 1 and 2
FGF3 c.166C>T, p.(Leu56Phe)
wt
hom
het
Family 3
GJB2 c.231G>A, p.(Trp77*)
hom
het
Family 4
 MYO7A c.470G>A, p.(Ser157Asn)
hom
het
Family 5
MYO7A c.3502C>T, p.(Arg1168Trp)
hom
het
Family 6
CDC14A c.1041dup, p.(Ser348Glnfs*2)
het
hom
Family 7
SLITRK6 c.120_121insT, p.(Asp41*)
c.1258A>T, p.(Asp41*)
c.1849T>C, p.(Ser617Pro)
c.4505A>G, p.(Asp1502Gly)
Family 8
MYO7A
c.1258A>T, p.(Asp41*)
c.1849T>C. p.(Ser617Pro)
c.4505A>G, p.(Asp1502Gly)
het
hom
Family 9
CDH23, c.2968G>A p.(Asp990Asn)
hom
hom
het
Family 10 / Family 11
GJB2, c.35delG p.(Gly12Valfs*2)
hom
het
Family 12
MYO15A, c.9518-2A>G
het
Family 13
CDH23, c.4688T>C
p.(Leu1563Pro)
Figure S1

## Slide 2
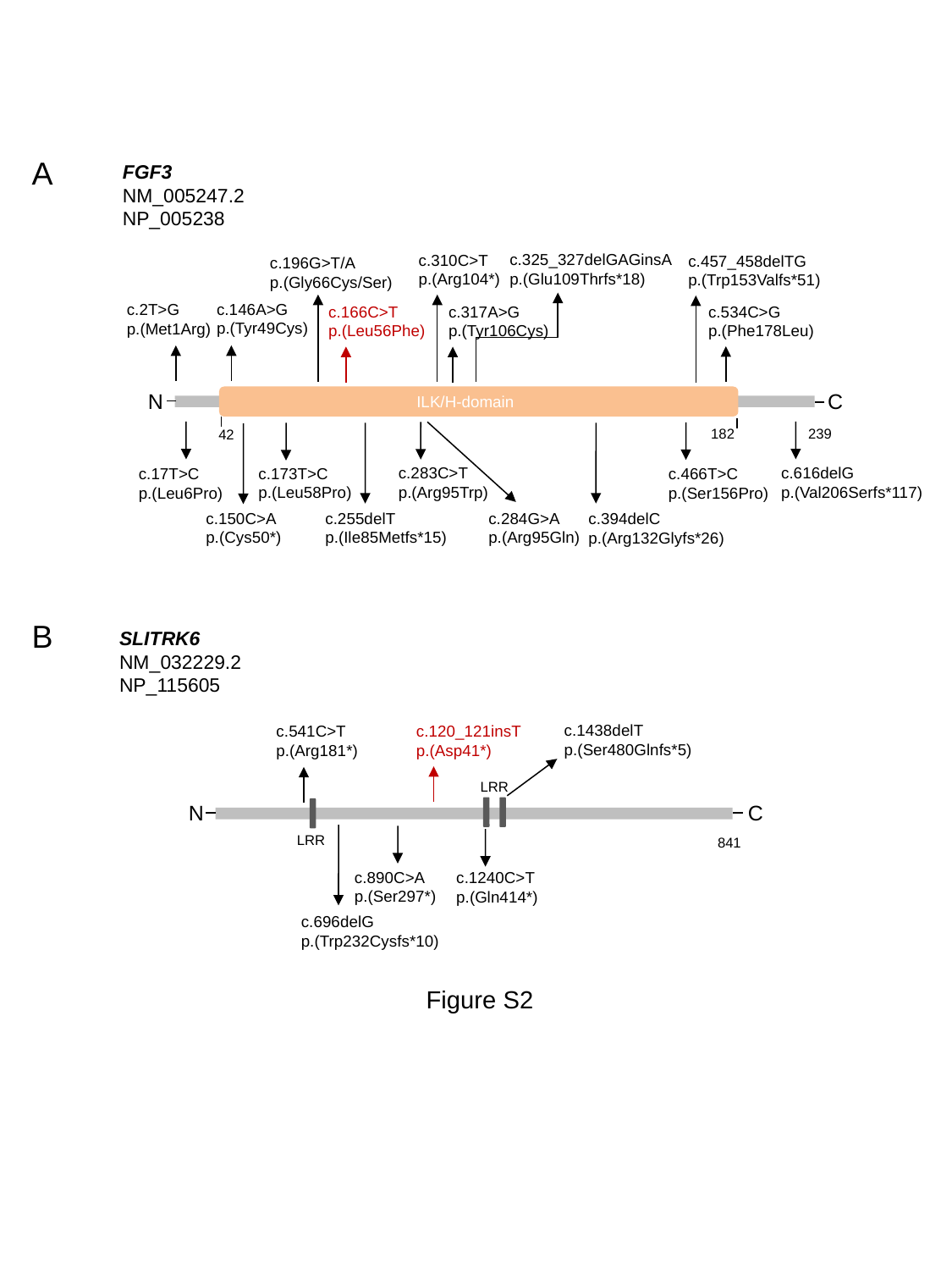

A
FGF3
NM_005247.2
NP_005238
c.325_327delGAGinsA
p.(Glu109Thrfs*18)
c.310C>T
p.(Arg104*)
c.457_458delTG
p.(Trp153Valfs*51)
c.196G>T/A
p.(Gly66Cys/Ser)
c.146A>G
p.(Tyr49Cys)
c.2T>G
p.(Met1Arg)
c.166C>T
p.(Leu56Phe)
c.534C>G
p.(Phe178Leu)
c.317A>G
p.(Tyr106Cys)
N
C
ILK/H-domain
182
239
42
c.616delG
p.(Val206Serfs*117)
c.283C>T
p.(Arg95Trp)
c.173T>C
p.(Leu58Pro)
c.17T>C
p.(Leu6Pro)
c.466T>C
p.(Ser156Pro)
c.255delT
p.(Ile85Metfs*15)
c.284G>A
p.(Arg95Gln)
c.150C>A
p.(Cys50*)
c.394delC
p.(Arg132Glyfs*26)
B
SLITRK6
NM_032229.2
NP_115605
c.1438delT
p.(Ser480Glnfs*5)
c.120_121insT
p.(Asp41*)
c.541C>T
p.(Arg181*)
LRR
N
C
LRR
841
c.890C>A
p.(Ser297*)
c.1240C>T
p.(Gln414*)
c.696delG
p.(Trp232Cysfs*10)
Figure S2
