# Supplementary material for: Genetic Spectrum of Syndromic and Non-Syndromic Hearing Loss in Pakistani Families
Source: Genes (Basel). 2020 Nov 11;11(11):1329. doi: 10.3390/genes11111329 (PMC7709052; doi:10.3390/genes11111329)
Supplement: Supplementary file 1 [file genes-11-01329-s001.zip › Supplementary Files/Table S1.docx]

**Table S1:** Primer sequences for identified variants in 13 Pakistani families with HL

| **Primer name** | **Sequence (5`-3`)** |
| --- | --- |
| *FGF3* c.166C>T For | CTCCTGGGTGGAAATTAAAGG |
| *FGF3* c.166C>T Rev | TCACTGTAGGCGCTGTTCT |
| *GJB2* c.23lG>A For | GATCTTCGTGTCCACGCCAGC |
| *GJB2* c.23lG>A Rev | CTTGACAGCTGAGCACGGGT |
| *MYO7A* c.470G>A For | AGAGCTTTCTAGAGTCAGAGTCTC |
| *MYO7A* c.470G>A Rev | ACAGCACAGAGTACATAGGTCTG |
| *MYO7A* c.3502C>T For | TAACTTTACCTGCCCTGTCCTCTCC |
| *MYO7A* c.3502C>T Rev | TATTTCTGTGGCCTTCAACTGAACC |
| *CDC14A* c.1041dup For | CTGAGGACTTCAGCAGTCAA |
| *CDC14A* c.1041dup Rev | AACTTGGTACTCGTGGCATC |
| *SLITRK6* c.120_121insT For | TCATGGTAATCCGTCATTTGCTT |
| *SLITRK6* c.120_121insT Rev | AGTTGTTTCAGGAGGCCAAGG |
| *MYO7A* c.1258A>T For | AGAGGGAACAGCTCAAGTAAAGG |
| *MYO7A* c.1258A>T Rev | GAGCCAACTAAATGTGCTTCG |
| *MYO7A* c.1849T>C For  *MYO7A* c.1849T>C Rev  *MYO7A* c.4505A>G For | ATCTTCCTGTAGGGTTCACTTCC  CACCCTACCTTATTCTTGACCTAGC  ACACTCTCTTCCTACTGCACTTTGG |
| *MYO7A* c.4505A>G Rev | ATGCCTCTTCACTTCCTGTGACC |
| *CDH23* c.2968G>A For | TCATTCCCTACTTGGTCTGG |
| *CDH23* c.2968G>A Rev | AGAGTGATCCAGCTGCAAAG |
| *GJB2* c.35delG For | GTTGTGTAAGAGTTGGTGTTTGC |
| *GJB2* c.35delG Rev | TTGTGTAGGTCCACCACAGGG |
| *MYO15A* c.9518-2A>G For | CAAATCCAGCTCTTAACCTCTAGC |
| *MYO15A* c.9518-2A>G Rev | ACCCTATGCTCACCAACATGG |
| *CDH23* c.4688T>C For | TCATTGCTCTGGGGTAGATG |
| *CDH23* c.4688T>C Rev | GCAGATGGACTAAAGGCAGA |
